# Antifungal Prophylaxis Utilization and the Associated Clinical Outcomes Among Pediatric Patients with Hematological Malignancies or Undergoing Hematopoietic Stem Cell Transplantation

**DOI:** 10.3390/jcm13237179

**Published:** 2024-11-26

**Authors:** Bushra Al Siyabi, Juhaina Salim Al-Maqbali, Dhanalekshmi Unnikrishnan Meenakshi, Yasir Wali, Laila Al Yazidi

**Affiliations:** 1 Department of Pharmacy, Sultan Qaboos University Hospital, University Medical City, Muscat 123, Oman; bushras@squ.edu.om; 2 Department of Pharmacology and Clinical Pharmacy, College of Medicine and Health Science, Sultan Qaboos University, Muscat 123, Oman; 3 College of Pharmacy, National University of Science and Technology, Muscat 130, Oman; dhanalekshmi@nu.edu.om; 4 Department of Child Health, College of Medicine and Health Sciences, Sultan Qaboos University, Muscat 123, Oman; yawali@squ.edu.om; 5 Department of Child Health, Sultan Qaboos University Hospital, University Medical City, Muscat 123, Oman; lailay@squ.edu.om

**Keywords:** antifungal prophylaxis, invasive fungal infection, children, hematological malignancies, HSCT, clinical outcome

## Abstract

**Background/Objectives:** Invasive fungal infections (IFIs) are a prevalent complication of intensive chemotherapy and hematopoietic stem cell transplantation (HSCT) in the pediatric population and are associated with high morbidity and mortality. We aimed to identify the utilization of antifungal prophylaxis prescriptions and the associated clinical outcomes. **Methods:** A retrospective study included children (≤18 years old) diagnosed with hematological malignancies or undergoing HSCT who are at high risk for developing IFI and received systemic antifungal therapy between January 2018 and April 2024 at Sultan Qaboos University Hospital (SQUH), Oman. **Results:** A powered sample of 222 patients was included, and 208 (93.69%) received antifungal prophylaxis. Among those who received prophylaxis, 148 (66.67%) received appropriate prophylaxis, 86.06% (*n* = 179) received appropriate dosage. The patients who did not receive antifungal prophylaxis had higher rates of inpatient IFI requiring treatment (85.71% versus 12.02%, *p* < 0.01), a longer median length of hospital stay (LOS) (67.5 days versus 10 days, *p* = 0.015), and more incidence of 90-day all-cause mortality (21.43% versus 2.88%, *p* < 0.01) than those who received antifungal prophylaxis. Survival analysis demonstrated that these patients had a 12% higher risk for earlier death. Also, being on antifungal prophylaxis reduces the odds of inpatient IFI requiring treatment, with an adjusted odds ratio (aOR) of 0.13 [95% CI: 0.019–0.801]. **Conclusions:** Antifungal prophylaxis utilization was high, and it markedly decreases the occurrence and enhances the prognosis of IFI. Nonetheless, inconsistencies in practice and a lack of pediatric-specific data underscore the necessity for uniform guidelines and additional research to strengthen preventative methods in this population, and proper TDM utilization could provide more robust insights.

## 1. Introduction

Invasive fungal infections (IFIs) are prevalent (6.04% to 25%) among children receiving intensive chemotherapy or undergoing hematopoietic stem cell transplantation (HSCT) [1,2,3,4]. They carry high rates of morbidity and mortality among immunocompromised patients [1]. There are several risk factors for developing IFIs in this population, which include intensive chemotherapy, prolonged neutropenia, increased use of glucocorticosteroids for graft vs. host disease (GVHD) [3], increasing age, and multiple and prolonged administration of broad-spectrum antibiotics [5,6].

The most common IFIs associated with this population include invasive aspergillosis and candidiasis, followed by mucormycosis and fusariosis [3,7,8]. In Oman, it was estimated that 79,520 people are affected by serious fungal infection each year, which accounts for 1.7% of the population, with an estimated 0.3% of the patients having invasive aspergillosis [9].

The implementation of antifungal prophylaxis as a preventive measure for IFI is currently being developed and is linked with improved outcomes [10,11,12,13,14]. A recent multicenter study from Australia demonstrated that the use of the appropriate mold-active prophylaxis in this population is associated with a reduction in the risk of IFI overall and increased IFI-free survival among children with AML [4].

The commonly used antifungal agents involves echinocandins, voriconazole, posaconazole, fluconazole and liposomal amphotericin [15]. However, the optimal dosage regimen is still poorly established [16]. Moreover, the appropriate use of antifungal agents depends on the type of malignancy, the chemotherapy used, and other patient’s related risk factors for prognosis [10,17]. Additionally, the need for therapeutic drug monitoring (TDM) for some of the antifungal agents with limited data on their pharmacokinetics makes it difficult to expedite the rate of cure and prevention of mortality, given the need for frequent adjustment and therapeutic range maintenance [18,19,20]. A study evaluating the appropriateness of antifungal agents in intensive-care units found that 47.3% of the antifungal regimen was prescribed inappropriately, and another study found that inappropriate antifungal dosing is common [21,22]. At the same time, a local study conducted at Sultan Qaboos University Hospital (SQUH) in Oman revealed that the overall use of antifungal agents in adult patients was inappropriately prescribed in 25.2% of the prescriptions [23]. Prescribing inappropriate antifungal regimens can lead to various significant consequences, including increased antifungal resistance, unnecessary exposure of patients to adverse outcomes associated with increased length of hospital stay (LOS), and potentially leading to treatment failure [23]. Resistance among Candida species is increasingly reported. Neonates in intensive care units (ICU) are at higher risk for these infections due to the widespread use of echinocandins and azoles. Similarly, resistance in Aspergillus fumigatus isolates has been linked to azole overuse and environmental factors, complicating treatment in immunocompromised children [24].

Pediatric patients present unique pharmacokinetic (PK) and pharmacodynamic (PD) challenges that complicate antifungal dosing, and studies looked into the appropriateness of the use of prophylaxis antifungal agents in the pediatric population are lacking, which makes it challenging to optimize the dosage of these agents [16]. Neonates and infants have immature hepatic and renal systems, leading to slower medication clearance and, therefore, risk of accumulation. On the contrary, older children may metabolize drugs faster than adults, requiring higher doses to achieve therapeutic concentrations. Furthermore, achieving these concentrations is critical, as small deviations in dosing can lead to subtherapeutic dosing or toxicity [25].

Furthermore, to the best of our knowledge, there were no local studies in the Omani pediatric population that investigated the appropriateness of antifungal use, although IFIs are common and serious, and the all-cause mortality among these high-risk patients remains high [1]. We aimed primarily to evaluate antifungal prophylaxis utilization in pediatric patients undergoing chemotherapy for hematological malignancies or post-HSCT measured by the indication, selection, dosage regimen, and monitoring parameters via TDM of the antifungal therapy used at Sultan Qaboos University Hospital (SQUH). Secondly, to assess the associated outcomes with IFI measured by LOS, all-cause mortality during admission, 90-day all-cause mortality, 90-day readmission, and 90-day occurrence of different types of IFI.

## 2. Methods

### 2.1. Study Design and Population

A retrospective cohort study was carried out using the data collected from the hospital’s electronic Trakcare system, including eligible patients admitted under the pediatric hematology/oncology team at Sultan Qaboos University Hospital (SQUH), University Medical City, Muscat, Oman, over a period between 1 January 2018, and 15 April 2024. All patients aged ≤18 years who were diagnosed with hematological malignancies or undergoing HSCT and who are at high risk for developing IFI, admitted under the pediatric hematology unit, and received oral or parenteral antifungal agents for treatment during a hospitalization were included, regardless of the stage of chemotherapy they are receiving during that episode on illness (at the induction stage, on other stages of chemotherapy, or not actively on chemotherapy). While patients with other hematological disorders or not admitted under the pediatric hematology unit or received topical or vaginal antifungal agents were excluded.

### 2.2. Definitions

Patients with a high risk for developing IFI were defined according to the 8th European Conference on Infections in Leukemia (ECIL-8) guideline [26]. Patients with acute myeloblastic leukemia (AML), recurrent acute leukemia, allogeneic HSCT, and acute lymphoblastic leukemia are at high risk (incidence rate of IFIs of >10%) and are eligible to receive antifungal prophylaxis.

The diagnoses of IFI in this population have been classified as proven, probable, or possible by the European Organization for Research and Treatment of Cancer/IFI Cooperative Group and the National Institute of Allergy and Infectious Diseases Mycoses Study Group Education and Research Consortium (EORTC/MSGERC) [27].

The assessment of the appropriateness of the indications, selection, dosage regimen, duration, and monitoring parameters of antifungal use will be carried out according to the recommendations of the Sultan Qaboos University Hospital (SQUH) pediatric infectious diseases team or according to the Sultan Qaboos University Hospital (SQUH) antifungal prophylaxis guideline for children with hematological malignancies or undergoing HSCT guideline, or according to ECIL-8 guideline, 2020 [26]. The appropriateness of antifungal prophylaxis is determined by adherence to the guidelines mentioned above, while it is deemed inappropriate when the guidelines are not followed. Regarding the timing of prophylaxis evaluation, the prophylaxis was assessed at all points of time during the hospital stay.

### 2.3. Sample Size

The sample size calculation was based on the main primary outcome: prevalence of prophylaxis antifungal use. A previous study by Ramírez et al. (2012) and his colleagues reported that antifungal use was indicated appropriately in 44.7% [22]. We hypothesize that the Sultan Qaboos University Hospital (SQUH) guideline is well-adherent in 80% of the studied population over the study period from 1 January 2018 to 15 April 2024. Therefore, using a power of 80% and an alpha level of 5%, the study requires 198 patients in total; we further incensed 222 patients to account for missing data.

### 2.4. Ethical Approval

This study was approved by the Medical Research Ethics Committee of the College of Medicine and Health Sciences of Sultan Qaboos University on 29 January 2024 (REF. NO. SQU-EC/009\2024, MREC # 3212).

### 2.5. Statistical Analysis

Categorical variables were presented as frequencies and percentages, whilst continuous variables were summarized using the mean and standard deviation (SD) for normally distributed variables and the median and interquartile range (IQR) for variables that are not normally distributed. To evaluate the associations between the groups, Mann–Whitney U test was used for abnormally distributed continuous variables. Similarly, Pearson’s χ^2^ test or Fisher’s exact test (when the cell number fell below 5) were used to assess the associations between the groups for categorical variables. All relevant variables or variables with a *p* < 0.05 were included in the backward stepwise multivariate logistic regression for categorical variables or mixed-effects linear regression for relevant continuous variables or variables with observed associations to find possible independent factors linked to different clinical outcomes (including patients’ age, gender, and weight. As well as patients with leukemia, who are at the induction stage or not actively on chemotherapy, on per-oral nutrition, admitted to isolation bed or ICU).

We performed a time-to-event survival analysis on 90-day all-cause mortality using the Kaplan–Meier method and complemented it with log-rank tests to assess the risk of all-cause mortality between the groups. The significance level for all tests was set at a two-tailed *p*-value below 0.05. The statistical analyses of the data were conducted using STATA version 18.0 (STATA Corporation, College Station, TX, USA).

## 3. Results

A total of 252 children with hematological malignancies and children undergoing HSCT were screened during the study period, among them, 30 patients were excluded for the following reasons: *n* = 15 patients had a standard risk (antifungal prophylaxis not required), *n* = 4 patients were admitted under hematology adult unit, and *n* = 11 patients had other hematological disorders.

In total, 222 pediatric patients were included in the study. The prevalence of antifungal prophylaxis prescriptions was 93.69% (208/222 patients). Females accounted for 39.19% of the total cohort, 38.94% of the antifungal prophylaxis group, and 42.86% of the non-prophylaxis group (*p* = 0.771). The groups categorized by prophylaxis status did not exhibit any statistically significant difference in terms of age and weight, as indicated by *p*-values of 0.217 and 0.076, respectively (Table 1).

Also, the groups were equally comparable in terms of the number of comorbidities and history of surgical experience, *p* = 0.876 and *p* = 0.377, respectively. Although the presence of two or more risk factors for IFI was higher in patients not on antifungal prophylaxis, this difference did not reach statistical significance (*p* = 0.074).

The nutritional source showed a notable disparity in oral feeding utilization, with a higher proportion in the antifungal prophylaxis group compared to the non-antifungal prophylaxis group (78.37% versus 50.00%, *p* = 0.015).

With regards to hematological condition type, most of the patients admitted post-HSCT were on IFI-prophylaxis, including the following: post-HSCT (immunological (13.94%), hemoglobinopathies (47.12%), leukemia (23.08%), and genetic disorders (3.37%)). Among the patients with acute leukemia, only 12.50% were on antifungal prophylaxis, while 85.71% of acute leukemia patients were not on antifungal prophylaxis (*p* < 0.01).

Regarding the chemotherapy stage, there was a considerably greater percentage of patients in the induction chemotherapy stage among those who did not receive antifungal prophylaxis (50.00% versus 6.73%; *p* < 0.01). The type of admission also showed significant variation, with a higher percentage of patients in the non-prophylaxis group admitted to the ICU (21.43% versus 4.81%; *p* = 0.039) and a lower proportion receiving isolation treatment (71.43% versus 89.90%; *p* = 0.034).

As shown in Table 1, the patients who did not receive antifungal prophylaxis had a longer median hospital stay compared to those who received prophylaxis (67.5 days versus 10 days, *p* = 0.015). Additionally, they had a far greater incidence of all types of inpatient IFI requiring treatment than patients on it (85.71% versus 12.02%, *p* < 0.01). However, there was no obvious difference in the incidence of definite IFI and duration of treatment between the two groups. Moreover, there was no significant difference in the distribution of any type of IFI—empirical, probable, or proven—between individuals receiving prophylaxis and those who were not.

The 90-day all-cause mortality rate showed a statistically significant difference between the groups, with more deaths found in patients who did not receive antifungal prophylaxis compared to those who were on antifungal prophylaxis (21.43% versus 2.88%, *p* < 0.01). At the same time, the hospital inpatient all-cause mortality rates did not reach a statistical difference between the groups (Table 1).

The findings of a multivariate regression analysis determined the association between independent variables and clinical outcomes are shown in Table 2. The analysis revealed that being on antifungal prophylaxis dramatically reduces the odds of inpatient IFI requiring treatment, with an adjusted odds ratio (aOR) of 0.13 and a 95% CI of 0.019 to 0.801 (*p* = 0.028). Additional independent factors associated with a lower risk of inpatient IFI requiring treatment are admission to an isolation bed and being on per-oral nutrition; both have significant protective aORs (*p* < 0.01).

Patients with leukemia were shown to have a substantially increased risk of IFI (aOR: 24.60; *p* < 0.01), indicating that leukemia is a strong predictor for the incidence of inpatient IFI.

For 90-day mortality, being on per-oral nutrition appeared to be a protective factor with a significant (aOR: 0.05; *p* < 0.01). Age showed an aOR of 0.05 per year increase and was also statistically significant with a *p*-value of 0.023.

The LOS was significantly longer for patients with leukemia and those requiring ICU admission, with aORs of 19.35 and 32.32, respectively, indicating a strong association between these factors and extended LOS.

**Figure 1:**
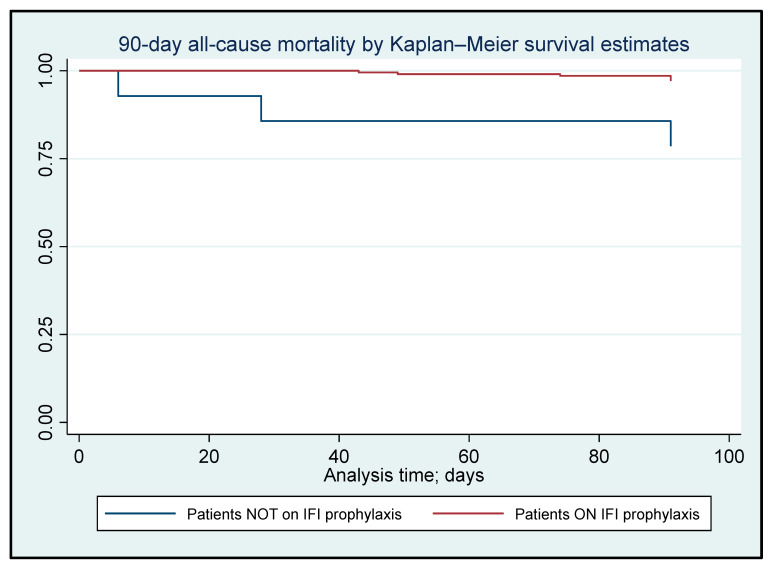
Kaplan–Meier analysis for the 90-day all-cause mortality classified according to the use of antifungal prophylaxis (N = 222). HR: 0.12, *p* < 0.01 [95% CI: 0.029–0.477].

Figure 1 shows the effect of antifungal prophylaxis on the 90-day all-cause mortality, the hazard ratio (HR) was 0.12 (95% CI: 0.029–0.477; *p* < 0.01), indicating that the group not on antifungal prophylaxis has a 12% higher risk of experiencing earlier death compared to the group on IFI.

Table 3 evaluates the appropriateness of the prescribing practices of the antifungal prophylaxis regimen for IFI prevention. Among the total of 222 patients, 148 (66.67%) were kept on appropriate antifungal prophylaxis (95% CI: 60.17–72.58%). At the same time, 74 (33.33%) were not on antifungal prophylaxis (95% CI: 27.41–39.83%).

Among the 208 patients who were on antifungal prophylaxis, 179 (86.06%) received a dosage regimen that was appropriately aligned with the recommended guidelines. However, only 29 patients (13.94%) were treated with inappropriate dosage.

In terms of the selection of IFI prophylaxis agents, 143 patients (68.75%) were administered a medication that aligned with the recommended guidelines, whereas 65 patients (31.25%) were given other types of antifungals outside the guideline recommendations.

Among 208, only a few patients had TDM monitoring completed, which indicated a low utilization of adequate monitoring (5.29%) due to the non-availability of the test in Oman. Nevertheless, as shown in Table 4, the assessment of TDM actions for 11 patients with measured TDM levels. The evaluation reveals that when TDM levels were found to be low, 5/5 of those patients (100%) had their doses changed appropriately (*p* < 0.015).

## 4. Discussion

This study is the first to provide such knowledge among pediatric patients with hematological malignancies or those undergoing HSCT in a tertiary hospital in Oman. Our findings showed that the prevalence of antifungal prophylaxis prescriptions was high, and the majority received an appropriate regimen. The study demonstrated that being on antifungal prophylaxis is an independent protective factor for the incident of inpatient IFI requiring treatment. Patients who did not receive antifungal prophylaxis had longer LOS and 90-day mortality rates. Also, survival analysis demonstrated that these patients had a higher risk of earlier death.

A recent study from Australia demonstrated that the burden of IFI among children is still high, particularly among children with high-risk acute lymphoblastic leukemia (ALL) compared to AML patients because of low uptake of antifungal prophylaxis among children with HR-ALL (56.7%) [4]. Our study showed that the prevalence of antifungal prophylaxis prescriptions was high (93.96%), and most of our patients who are at high risk of IFI received the appropriate regimen (*n* = 148; 66.67%) in terms of agent selection and dosing, which is higher than what was reported in some studies where the inappropriateness rate was close to 50% or more [24,25]. The hospital experience of prescribing IFI prophylaxis at SQUH improved significantly following the development of the hospital guidelines in 2019 by the infectious control monitoring team. Invasive candidiasis and mold infections carry a high all-cause mortality rate in children with hematological malignancies and those undergoing HSCT. One study reported the all-cause mortality rate to be over 20% and over 50%, respectively [28]. Lehrnbecher T and his colleagues [29] highly recommended using mold active agents for pediatric patients at high risk of obtaining IFI in their systematic review, which assessed the efficacy of giving antifungal prophylaxis for these patients.

IFI lowers the survival rate significantly in children with hematological malignancies (38.8%) compared to those without IFIs (69.9%) [30]. K. Czyżewski et al. reported that the introduction of a national antifungal prophylaxis program in their setting significantly decreased the incidence of IFI among children after HSCT and malignancy treatment, which resulted in improved outcomes. The antifungal prophylaxis resulted in decreased incidence of IFD in patients undergoing HSCT (from 23.1% to 13.4%) and children with AML on conventional chemotherapy (from 36% to 23%) [31]. This study found that patients who did not receive antifungal prophylaxis had a far greater incidence of all types of inpatient IFI requiring treatment. In other words, we demonstrated that being on antifungal prophylaxis is an independent protective factor for the incident of inpatient IFI requiring treatment. This underlines the essential role of prophylaxis in preventing IFIs in hospitalized patients, particularly in those at higher risk. Our findings are consistent with those of previous studies that demonstrated the effectiveness of antifungal prophylaxis in reducing the incidence of IFIs in specific patient populations [4,32].

Additionally, the study found a notable link between leukemia and admission to the ICU and prolonged LOS. These results were reflected in a previous study, which found that the need for ICU admission is high in patients with acute lymphoblastic leukemia (ALL) [33,34]. This highlights the significant healthcare burden of leukemia complications. Given these findings, early detection and aggressive management might reduce the negative outcomes.

There has been conflicting evidence in the literature on the effects of receiving antifungal prophylaxis on all-cause mortality and the occurrence of IFI in post-HSCT children and those with malignancies. Some studies showed a reduction in all-cause mortality because of reducing the rates of IFI [31,32]. Yeoh Y. and his colleagues [4], in their recent study, demonstrated increased IFI-free survival among children with AML but not those with HR-ALL. They related that to a higher antifungal prophylaxis uptake among children with AML (98.7%) compared to those with high-risk ALL (56.7%). This study demonstrated higher 90-day all-cause mortality rates among patients with a high risk of IFI who did not receive antifungal prophylaxis. In addition, the survival analysis demonstrated that these patients had a higher risk of earlier death (HR: 0.12). Antifungal prophylaxis has been shown to decrease all-cause mortality in various patient groups. For instance, in cancer patients’ post-chemotherapy, prophylaxis reduced mortality significantly (RR, 0.84; 95% CI, 0.74 to 0.95). Also, in allogeneic HSCT recipients, prophylaxis reduced all-cause mortality (RR, 0.62; 95% CI, 0.45 to 0.85) and fungal-related mortality. Furthermore, prophylaxis significantly reduced fungal-related mortality and documented IFI, although the reduction in overall mortality was borderline significant (RR, 0.88; 95% CI, 0.74 to 1.06) [35]. The associations could be attributed to several factors, such as the overall burden of infection or complications arising from delayed treatment; hence, it highlights the potential long-term benefits of starting prophylaxis for patients at high risk of developing IFI [36].

Moreover, it is essential to note that the study did not demonstrate a statistical difference in hospital inpatient mortality rates, 90-day admissions with IFI, or 90-day admissions with other infections. This may be due to the study sample size or other confounding factors that influence in-hospital mortality. Several studies have been conducted on the association between the development of IFI and mortality [37], but not the association between IFI prophylaxis administration and mortality. Further research with a larger sample size that accounts for potential more confounders is needed to confirm these findings and to further explain the relationship between IFI prophylaxis, treatment outcomes, and mortality.

This study offers a thorough analysis to identify predictors of the outcomes associated with receiving antifungal prophylaxis and was the first to demonstrate a time-to-event survival analysis and showed that receiving antifungal prophylaxis can potentially delay death. However, it was limited by its retrospective design, and the data were restricted to the information provided in the medical records, and episodes were selected randomly regardless of the time of the admission, whether it is before, within, or after engrafting for some of the patients. This was a single-center study with a relatively small sample size and heterogeneous patient groups. The limited TDM testing facility in our center may have affected the overall effectiveness of utilizing antifungal prophylaxis, and it introduced variability in treatment efficacy and safety outcomes. Future studies with TDM could provide more robust insights. Additionally, variability in clinical practice and adherence to guidelines across hospitals in Oman can influence outcomes. Future prospective studies should be conducted to support these findings and to improve the use of prophylactic antifungal strategies in pediatric hematology/oncology and post-HSCT settings in Oman.

## 5. Conclusions

Antifungal prophylaxis utilization in the study setting was high, and it markedly decreased the occurrence and enhanced the prognosis of IFI in pediatric patients with hematological malignancies and those undergoing HSCT. Nonetheless, inconsistencies in practice and a lack of pediatric-specific data, in general, underscore the necessity for uniform guidelines and additional research to enhance preventative methods in these high-risk populations. Additionally, future studies with proper TDM utilization could provide more robust insights into prophylaxis efficacy and safety outcomes.

## Figures and Tables

**Table 1:** Characteristics, clinical features, and clinical outcomes of the admitted patients classified according to the use of antifungal prophylaxis (*n* = 222).

Characteristic *n* (%) Unless Specified Otherwise	Total 222 (100%)	On Antifungal Prophylaxis 208 (93.69%)	NOT on Antifungal Prophylaxis 14 (6.31%)	*p*-Value
Female sex	87 (39.19%)	81 (38.94%)	6 (42.86%)	0.771
Age; IQR, years	6 (3–9)	6 (3–9)	8 (3–12)	0.217
Weight; IQR, kg	16 (11–23)	16 (11–22)	23 (13–34)	0.076
Medical history				
Presence of comorbidities	44 (19.82%)	41 (19.71%)	3 (21.43%)	0.876
Presence of surgeries	11 (4.95%)	11 (5.29%)	0	0.377
Presence of ≥2 risk factors for IFI	183 (82.43%)	169 (81.25%)	14 (100%)	0.074
Nutritional source				
Per oral	170 (76.58%)	163 (78.37%)	7 (50.00%)	0.015
Per oral plus formula	5 (2.25%)	4 (1.92%)	1 (7.14%)	0.280
NGT	14 (6.31%)	12 (5.77%)	2 (14.29%)	0.217
TPN	33 (14.86%)	29 (13.94)	4 (28.57%)	0.136
Type of hematological conditions				
Post-BMT (immunological)	29 (13.06%)	29 (13.94%)	0	0.225
Post-BMT (hemoglobinopathies)	98 (44.14%)	98 (47.12%)	0	<0.01
Post-BMT (leukemia)	49 (22.07%)	48 (23.08%)	1 (7.14%)	0.314
Post-BMT (genetic disorders)	8 (3.60%)	7 (3.37%)	1 (7.14%)	0.411
High risk acute leukemias	38 (17.12%)	26 (12.50%)	12 (85.71%)	<0.01
Stage of chemotherapy during hospitalization				
On induction chemotherapy	21 (9.46%)	14 (6.73%)	7 (50.00%)	<0.01
Not actively on chemotherapy	154 (69.37%)	151(72.60%)	3 (21.43%)	<0.01
All other chemotherapy stages	47 (21.17%)	43 (20.67%)	4 (28.57%)	0.502
Type of admission				
Normal bed	7 (3.15%)	7 (3.37%)	0	1.000
Isolation	197 (88.74%)	187 (89.90%)	10 (71.43%)	0.034
High Dependency Unit (HDU)	5 (2.25%)	4 (1.92%)	1 (7.14%)	0.280
Intensive Care Unit (ICU)	13 (5.86%)	10 (4.81%)	3 (21.43%)	0.039
Clinical outcomes				
Length of stay (LOS), IQR, days	13 (2–50)	10 (2–48)	68 (41–113)	<0.01
Incidence of inpatient IFI (definite)	8 (21.62%)	4 (16.00%)	4 (33.33%)	0.394
Incidence of inpatient IFI requires treatment (ALL)	37 (16.67%)	25 (12.02%)	12 (85.71%)	<0.01
Duration of IFI treatment, IQR, days	17 (10–35)	15(10–33)	23 (10–41)	0.626
Type of IFI				
Empirical IFI	27 (72.97%)	19 (76.00%)	8 (66.67%)	0.550
Probable IFI	5 (13.51%)	2 (16.67%)	3 (12.00%)	1.000
Possible IFI	2 (5.41%)	2 (11.75%)	0	0.204
Proven IFI	3 (8.11%)	1 (4.00%)	2 (16.67%)	0.241
Inpatient all-cause mortality	4 (1.80%)	3 (1.44%)	1 (7.14%)	0.231
90-day all-cause mortality	9 (4.05%)	6 (2.88%)	3 (21.43%)	<0.01
90-day admission with IFI	2 (0.90%)	2 (0.96%)	0	1.000
90-day admission with other all-type of infections	92 (42.44%)	86 (41.35%)	6 (42.86%)	1.000

**Table 2:** Multivariate regression for independent factors associated with the use of antifungal prophylaxis and clinical outcomes.

Outcome Tested in the Model (Dependent Factor)	Independent Factors	aOR * [95% CI]	*p*-Value
Incidence of inpatient IFI required treatment *	On antifungal prophylaxis	0.13 [0.019–0.801]	0.028
	With leukemia	24.60 [5.698–106.216]	<0.01
	Admitted to isolation bed	0.13 [0.033–0.488]	<0.01
	On per-oral nutrition	0.05 [0.013–0.178]	<0.01
90-day all-cause mortality *	On per-oral nutrition	0.05 [0.013–0.178]	<0.01
	Age; year	0.05 [0.013–0.178]	0.023
	Weight; kg	1.15 [0.033–0.178]	0.033
Length of stay (LOS); days **	With leukemia	19.35 [2.79–35.923]	0.022
	Intensive Care Unit (ICU) admission	32.32 [12.459–56.198]	<0.01

* Backward stepwise binary multivariate logistic regression model for an adjusted odds ratio (aOR) for the following relevant factors that include patients’ age, gender, and weight. As well as patients with leukemia who are at the induction stage or not actively on chemotherapy, on per-oral nutrition, or admitted to isolation bed or ICU. ** Backward stepwise mixed-effects linear regression model for an adjusted odds ratio (aOR) for the following relevant factors that include patients’ age, gender, and weight. As well as patients with leukemia who are at the induction stage or not actively on chemotherapy, on per-oral nutrition, or admitted to isolation bed or ICU.

**Table 3:** Assessment of antifungal prophylaxis prescribing appropriateness based on the guideline according to addition of the drug, the dosage, the selection, and the TDM monitoring.

Characteristic *n* (%) Unless Specified Otherwise	Total	Appropriate	95% CI	Inappropriate	95% CI
Addition of prophylaxis	222 (100%)	148 (66.67%)	60.17–72.58%	74 (33.33%) *	27.41–39.83%
Dosage	208 (100%)	179 (86.06%)	80.62–90.16%	29 (13.94%)	9.84–19.38%
Selection		143 (68.75%)	62.09–74.71%	65 (31.25%)	25.29–37.91%
TDM monitoring		11 (5.29%)	3.29 (9.91%)	197 (94.71%)	90.01–96.71%

* Inappropriate non-addition of antifungal prophylaxis was seen in 14 (100%) patients versus 60 (33.33%) patients who were inappropriately added to antifungal prophylaxis (*p* < 0.01).

**Table 4:** Assessment of the actions on TDM monitoring for patients with TDM levels (*n* = 11).

Characteristic *n* (%) Unless Specified Otherwise	Total 11 (100%)	Dose Changed 5 (45.45%)	Dose Unchanged 6 (54.55%)	*p*-Value
Low level	6 (54.55%)	5 (100.00%)	1 (16.67%)	0.015
In-range level	5 (45.45%)	0	5 (83.33%)	0.015
High level	0	0	0	-

## Data Availability

Data are available from the corresponding author on request.
